# Antiviral activity of lambda-carrageenan against influenza viruses and severe acute respiratory syndrome coronavirus 2

**DOI:** 10.1038/s41598-020-80896-9

**Published:** 2021-01-12

**Authors:** Yejin Jang, Heegwon Shin, Myoung Kyu Lee, Oh Seung Kwon, Jin Soo Shin, Yong-il Kim, Chan Woo Kim, Hye-Ra Lee, Meehyein Kim

**Affiliations:** 1 Infectious Diseases Therapeutic Research Center, Korea Research Institute of Chemical Technology (KRICT), 141 Gajeongro, Yuseong, Daejeon, 34114 Republic of Korea; 2 Department of Chemistry, Korea Advanced Institute of Science and Technology (KAIST), Daejeon, 34141 Republic of Korea; 3 Hanmi Pharmaceutical Co., Hwaseong-si, Gyeonggi-do 18536 Republic of Korea; 4 Department of Biotechnology and Bioinformatics, College of Science and Technology, Korea University, Sejong, 30019 Republic of Korea; 5 Graduate School of New Drug Discovery and Development, Chungnam National University, Daejeon, 34134 Republic of Korea; 6 Present Address: siRNAgen Therapeutics Co., Daejeon, 34302 Republic of Korea

**Keywords:** Drug discovery, Microbiology

## Abstract

Influenza virus and coronavirus, belonging to enveloped RNA viruses, are major causes of human respiratory diseases. The aim of this study was to investigate the broad spectrum antiviral activity of a naturally existing sulfated polysaccharide, lambda-carrageenan (λ-CGN), purified from marine red algae. Cell culture-based assays revealed that the macromolecule efficiently inhibited both influenza A and B viruses with EC_50_ values ranging from 0.3 to 1.4 μg/ml, as well as currently circulating severe acute respiratory syndrome coronavirus 2 (SARS-CoV-2) with an EC_50_ value of 0.9 ± 1.1 μg/ml. No toxicity to the host cells was observed at concentrations up to 300 μg/ml. Plaque titration and western blot analysis verified that λ-CGN reduced expression of viral proteins in cell lysates and suppressed progeny virus production in culture supernatants in a dose-dependent manner. This polyanionic compound exerts antiviral activity by targeting viral attachment to cell surface receptors and preventing virus entry. Moreover, its intranasal administration to mice during influenza A viral challenge not only alleviated infection-mediated reductions in body weight but also protected 60% of mice from virus-induced mortality. Thus, λ-CGN could be a promising antiviral agent for preventing infection with several respiratory viruses.

## Introduction

**Figure 1:**
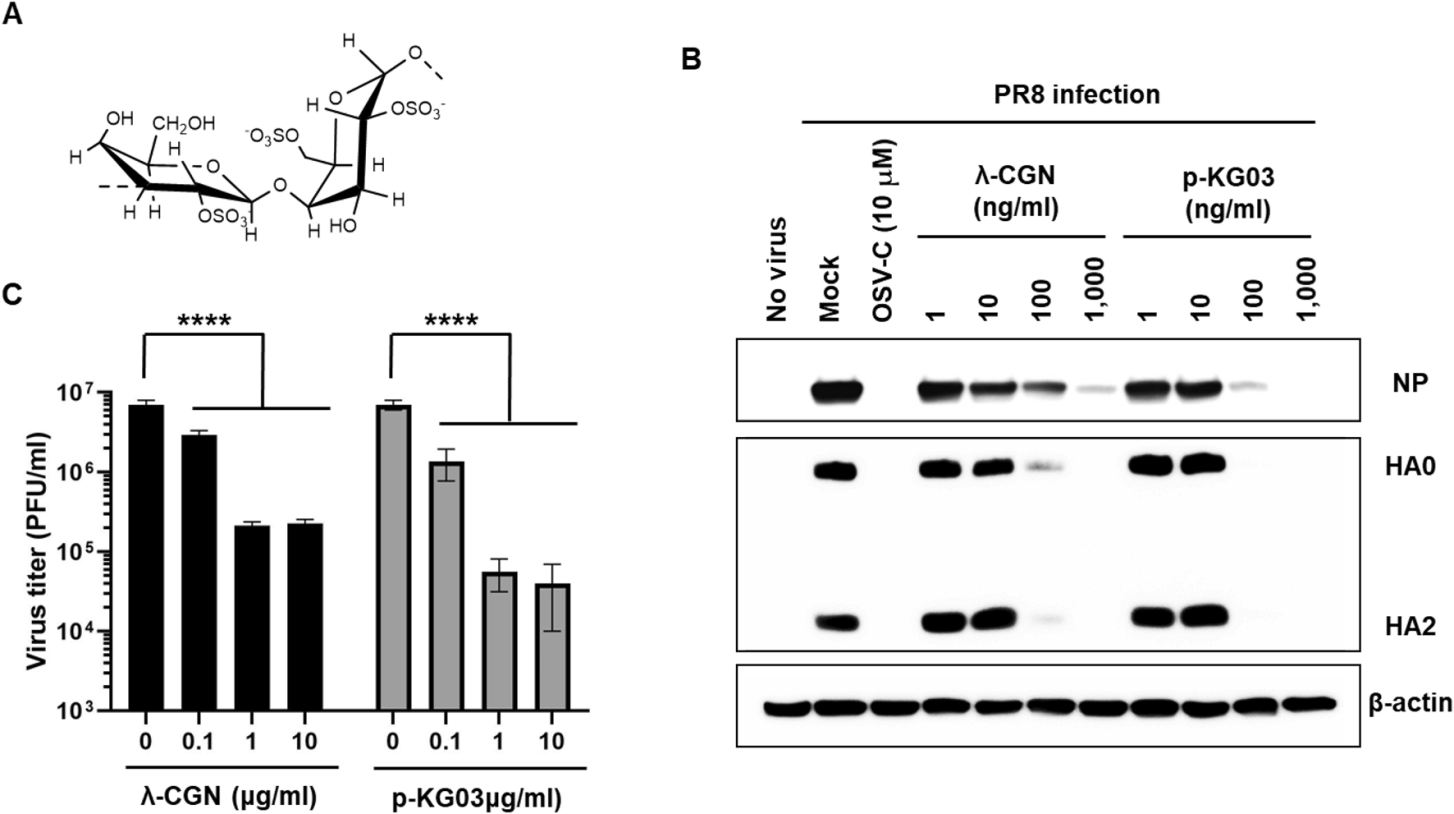
Inhibition of influenza virus infection by λ-CGN in vitro. (**A**) Chemical structure of the repeating disaccharide unit in λ-CGN. (**B**) Western blot analysis showing expression of viral proteins. MDCK cells infected with PR8 at an MOI of 0.001 were mock-treated (Mock) or treated with 10 μM of OSV-C or with increasing concentrations of λ-CGN or p-KG03 at 35 °C. On the next day, cell lysates were harvested for SDS-PAGE and immunoblotting with anti-NP or anti-HA antibodies. β-Actin was used as a loading control. ‘No virus’ means negative control without viral infection. Proteins are indicated on the right side of the panels. (**C**) Plaque assay to determine viral titers. Serial ten-fold dilutions of cell culture supernatants acquired after viral infection and compound treatment in (**B**) were loaded onto fresh MDCK cells and cultured at 33 °C in 1.2% Avicel-containing overlay medium. The number of viral plaques was counted after crystal violet staining on day 3 post-infection. Data are expressed as the mean ± S.D. of three independent experiments. ****P < 0.0001. The graph was created using GraphPad Prism 8.3.1 (www.graphpad.com).

Carrageenans (CGNs) extracted from marine seaweeds belong to a family of sulfated D-series polysaccharides harboring α-galactose residues. The diverse chemical structure and the degree of sulfation divides CGNs into three major polysaccharide groups, kappa (κ)-, iota (ι)- and lambda (λ)-CGNs, which contain one, two, and three negatively-charged sulfate ester groups per disaccharide repeating unit, respectively^1^. These natural polymers of diverse molecular weights have been used widely as pharmaceutical delivery vehicles to facilitate drug formulation or sustained drug release. As biomolecules, CGNs have various biological activities, including anticoagulant, anti-tumoral, or immunomodulatory functions^2,3^. Several reports suggest that CGNs show in vitro or in vivo activity against rhinovirus, enterovirus 71, dengue virus, human herpes simplex, African swine fever virus, and influenza A virus^4–10^. Most of these antiviral efficacy studies have focused on κ- and ι-CGNs; only one study suggested that λ-CGN was a potent inhibitor of rabies virus infection^11^. Based on the structural characteristics of λ-CGN, by which it has no 3,6-anhydro-d-galactopyranosyl linkage but contains a higher sulfate content compared to the two other sulfated polysaccharides (Fig. 1A), we wondered whether it is active against different respiratory viruses: influenza A and B viruses and severe respiratory syndrome coronavirus 2 (SARS-CoV-2).

Influenza virus is a major human respiratory virus that causes seasonal epidemics or unexpected pandemic outbreaks. It belongs to the family *Orthomyxoviridae* and contains an eight-segmented, negative-sense RNA genome classified into three types, A, B and C. Type A is further divided into subtypes based on the serological characteristics of surface glycoproteins hemagglutinin (HA) and neuraminidase (NA), while type B is split into Victoria and Yamagata lineages. Even though therapeutic antivirals such as oseltamivir phosphate, zanamivir, peramivir and baloxavir marboxyl, as well as preventative vaccines, have been successfully developed, emerging drug-resistant strains and mismatch-derived inefficacy of vaccines mean that this virus still threatens human public health, resulting in an estimated annual mortality burden of 290,000 to 650,000 deaths^12–14^.

Coronavirus, a member of the family *Coronaviridae*, is also an enveloped virus with a positive-sense single-stranded RNA genome of 26 to 32 kilobases in length. Similar to influenza virus, it is a zoonotic virus that causes respiratory disease in humans. Most infections have been related to mild symptoms such as fever, fatigue, or dry cough. However, recently emerging viruses have become more lethal and highly contagious. For example, SARS-CoV, first identified in 2003, had a mortality rate of 10%, with over 8000 laboratory-confirmed cases, whereas Middle East respiratory syndrome coronavirus (MERS-CoV), identified in 2012, had a mortality rate of 34%, with 2494 cases^15,16^. In comparison to SARS-CoV and MERS-CoV, currently circulating SARS-CoV-2 has a lower fatality rate (about 9% compared with SARS-CoV-1); however, it has caused a global pandemic, with over forty-four million confirmed cases and 1178 thousand deaths as of November 2020 recorded since December 2019^17^. Despite this formidable circulation, we still have no coronavirus-specific antivirals. Because the symptoms and transmission routes of these respiratory viruses are very similar, a broad spectrum antiviral agent is required for their co-treatment. Therefore, the aim of this study was to assess the antiviral activity of λ-CGN against influenza viruses and SARS-CoV-2 and to identify its mechanism of action.

## Experimental section

### Cells, viruses, and compounds

Madin–Darby canine kidney (MDCK) cells, African green monkey kidney cells (both Vero and Vero E6) and human embryonic kidney (HEK) 293 T cells were purchased from the American Type Culture Collection (Cat. Nos., CCL-34, CCL-81, CRL-1586 and CRL-3216, respectively; ATCC, Manassas, VA, USA). MDCK cells were maintained in minimum essential medium (MEM; HyClone, Logan, UT, USA) supplemented with 10% fetal bovine serum (FBS; Atlas Biologicals, Fort Collins, CO, USA). Other cell lines were maintained in Dulbecco’s modified Eagle’s medium (DMEM; HyClone) supplemented with 10% FBS. Influenza viruses A/Puerto Rico/8/34 (PR8; H1N1), A/Hong Kong/8/68 (HK; H3N2), and B/Lee/40 (Lee) were purchased from the ATCC. The mouse-adapted PR8 (maPR8) strain was a kind gift from Prof. H. J. Kim (Chung-Ang University, Seoul, Republic of Korea). Influenza A viruses were inoculated into 10-day-old embryonated chicken eggs at 37 °C for 3 days, whereas influenza B virus was amplified at 35 °C for 3 days in MDCK cells in the presence of 2 μg/ml tosyl phenylalanyl chloromethyl ketone (TPCK)-treated trypsin (Sigma-Aldrich, St. Louis, MO, USA). SARS-CoV-2 (BetaCoV/Korea/KCDC03/2020), provided by Korea Centers for Disease Control and Prevention, was amplified in Vero cells at 37 °C for 3 days. After centrifugation at 1000*g* for 5 min, viral stocks were stored at − 80 °C and viral titers were determined in a plaque assay^18^. The test compound λ-CGN, average molecular weight 1025 kDa, was purchased from DuPont Nutrition & Biosciences (Wilmington, DE, USA). Control anti-influenza viral agents amantadine hydrochloride (AMT; ≥ 98%), ribavirin (RBV; ≥ 98%) and (–)-epigallocatechin gallate (EGCG; ≥ 95%) were purchased from Sigma-Aldrich. Oseltamivir carboxylate (OSV-C) was purchased from United States Biological (Swampscott, MA, USA). Marine microalgae-derived sulfated polysaccharide p-KG03 was purified and characterized by Dr. Joung Han Yim (Korea Polar Research Institute, Incheon, Republic of Korea)^19^. Oseltamivir phosphate (OSV-P; ≥ 98%) for in vivo antiviral studies was obtained from Hanmi Pharmaceutical Co. (Gyeonggi-do, Republic of Korea). Remdesivir (RDV; 99.74%), a control anti-SARS-CoV-2 compound, was purchased from MedChem Express (Monmouth Junction, NJ, USA).

### Cell culture-based antiviral assay

An antiviral assay for influenza viruses was performed as described previously^20^. Briefly, MDCK cells grown overnight in 96-well plates (3 × 10^4^ cells per well) were mock-infected or infected with each viral strain at a multiplicity of infection (MOI) of 0.001 at 35 °C for 1 h. After removing unabsorbed virus, cells were treated with threefold dilutions of each compound for 3 days at the same temperature. Viability of non-infected or infected cells was measured using 3-(4,5-dimethylthiazol-2-yl)-2,5-diphenyltetrazoliumbromide (MTT) to determine the half-maximal cytotoxic concentration (CC_50_) and the half-maximal effective concentration (EC_50_), respectively.

To assess anti-SARS-CoV-2 activity in an image-based system, Vero cells were grown overnight in 96-well plates (2 × 10^4^ cells per well). After addition of serial threefold dilutions of compounds, cells were infected with an equal volume of SARS-CoV-2 (MOI of 0.05) at 37 °C for 2 days within a biosafety level 3 laboratory. The cells were fixed and permeabilized with chilled acetone:methanol (1:3) for probing with an anti-spike antibody (Genetex, Irvine, CA, USA) followed by Alexa Fluor 488-conjugated goat anti-mouse IgG (Invitrogen, Carlsbad, CA, USA) to determine EC_50_ values. Cell nuclei were counterstained with 4′,6-diamidino-2-phenylindole (DAPI; Invitrogen) to calculate the CC_50_ values. The number of viral spike protein-derived or cell nuclei-derived signals detected in four spots per well was quantified from three independent samples using the Operetta high content screening system (Perkin Elmer, Waltham, MA, USA) and the built-in Harmony High-Content Imaging and Analysis software 3.5.2. For determination of 50% tissue culture infectious dose (TCID_50_), SARS-CoV-2-infected cells were incubated in the absence or presence of antiviral compounds for 2 days. Fresh Vero cells seeded in 96-well plates were infected with serial tenfold dilutions of the culture supernatants for additional 2 days. TCID_50_s were determined by counting SARS-CoV-2 spike protein-derived green fluorescence population as well as the number of DAPI-derived nuclei distribution as mentioned above.

### Western blot analysis

For anti-influenza viral study, PR8-infected MDCK cells (MOI, 0.001) were treated with increasing concentrations of λ-CGN, pKG-03 or OSV-C at 35 °C for 1 day. For antiviral analysis against SARS-CoV-2, virus-infected Vero cells (MOI, 0.005) were treated with increasing concentrations of λ-CGN or RDV at 37 °C for 2 days. Culture lysates were harvested and loaded onto 10 or 12% SDS-PAGE gels (40 μg total protein per well) for electrotransfer. Influenza viral NP and HA proteins were detected using mouse anti-NP (Cat. No., 11675-MM03; Sino Biological, Beijing, China) and rabbit anti-HA2 (Cat. No., 86001-RM01; Sino Biological) antibodies, respectively, according to our previous report^18^. SARS-CoV-2 spike protein was probed with the mouse anti-spike antibody. Cellular β-actin used as a loading control was detected with a mouse anti-β-actin antibody (Cat. No., A1987; Sigma-Aldrich). Horseradish peroxidase (HRP)-conjugated goat anti-mouse or anti-rabbit secondary antibodies were used to detect the primary antibodies (Thermo Scientific, Waltham, MA, USA). After addition of a chemiluminescent HRP substrate (SuperSignal West Pico Chemiluminescent Substrate; Pierce, Rockford, IL, USA), images were obtained using a LAS-4000 Luminescent Image Analyzer (Fujifilm, Tokyo, Japan).

### Plaque titration

Plaque reduction assay was performed as described previously, with some modifications^18^. Briefly, MDCK cells seeded in 6-well plates were infected with PR8 at an MOI of 0.001 in the absence or presence of increasing concentrations of λ-CGN or p-KG03. On the next day, the culture supernatants were harvested and serial tenfold dilutions were used for infection of fresh MDCK cells in 48-well plates. After their incubation in overlay medium [serum-free MEM with 1.2% Avicel RC-591 (FMC Corp, Philadelphia, PA, USA) and 2 μg/ml TPCK-trypsin (Sigma-Aldrich)] at 33 °C for 3 days, the number of plaques was counted by crystal violet staining.

### Time-of-addition experiments

MDCK cells were seeded in 48-well plates at a density of 1.5 × 10^5^ cells per well for 1 day. In the pre-treatment assay, λ-CGN (1 μg/ml) was treated for 2 h followed by PBS-washing and infection with PR8 at an MOI of 0.001 for additional 2 h. In the co-treatment assay, the mixture of λ-CGN and PR8 were loaded onto the cells immediately or after 30-min-preincubation. In the post-treatment assay, λ-CGN was added for 2 h after infection with PR8 for 2 h. As controls, p-KG03 (1 μg/ml) or EGCG (1 μM) was treated in all sets. In each step, cells were washed with PBS to remove non-specifically bound compounds or unabsorbed virus. The cells were cultured at 33 °C for 3 days in the overlay medium for plaque titration^19^.

### HA inhibition assay

Equal volumes (25 μl) of two-fold dilutions of PR8 (HA titer of the stock, 2^8^) and increasing concentrations of λ-CGN were incubated for 20 min at room temperature in 96-well round bottom plates. In each well, 50 μl of 0.5% chicken red blood cells (RBCs) was added. After 30 min incubation, HA titers were determined by RBC agglutination^21^.

### Confocal microscopy

MDCK cells were infected with PR8 virus at an MOI of 5 in the absence or presence of the sulfated polysaccharides (10 μg/ml) for 4 h at 37 °C. In parallel, the same samples were incubated for 2.5 h at 37 °C with protein synthesis inhibitor cycloheximide (10 μg/ml) (CHX; Sigma-Aldrich), of which experimental condition was optimized in our previous reports^18,22^. Viral NP was visualized using an anti-NP antibody (Cat. No., sc-80481; Santa Cruz Biotechnology, Santa Cruz, CA, USA) and Alexa Fluor 488-conjugated goat anti-mouse IgG (Invitrogen), while nuclear DNA was counterstained using 4′,6-diamidino-2-phenylindole (DAPI; Vector Laboratories, Burlingame, CA, USA). Images were captured under a Zeiss LSM 700 confocal microscope and data were analyzed with ZEN blue software 3.1 (Carl Zeiss, Thornwood, NY, USA).

### In vivo study

Antiviral efficacy study in a mouse model was performed by modification of our previous report^18^. Briefly, female BALB/c mice (6–7 weeks old; Orient Bio Inc., Gyeonggi-do, Republic Korea) were infected with maPR8. Five units of 50% mouse lethal dose (5 MLD_50_) of the virus were preincubated with λ-CGN for 30 min at room temperature. Mice were challenged intranasally with maPR8 alone or with maPR8 mixed with λ-CGN (1 or 5 mg/kg) in a total volume of 50 μl. The control group received OSV-P orally from days 0 to 5 post-infection [10 mg/kg/day (b.i.d.)] beginning 4 h before virus challenge. Changes in body weight and mortality were measured every day for 15 days. Mice were sacrificed when they lost at least 25% of their body weight. All animal experiments were conducted in accordance with ethical guidelines approved by the Institutional Animal Care and Use Committee (IACUC) of the Korea Research Institute of Chemical Technology (KRICT). All experimental protocols were approved by the KRICT’s IACUC with the code number of 2020-6D-04-01. Kaplan–Meier survival curves were constructed using GraphPad Prism 8.3.1 (GraphPad Software, San Diego, CA, USA).

### Pseudovirus production and neutralization assay

Pseudotyped viruses were prepared by transfecting 293T cells with following plasmids: encoding HIV-1 genome (gag/pol) together with glycoprotein(s) of each virus (pCAGGS-Influenza A (H1N1) HA together with pCAGGS-Influenza A (H1N1) NA, or pCAGGS-SARS-CoV-2-spike), and the firefly luciferase (pQCXIP-fLuc). Transfection was performed with PEIpro (Polyplus-transfection, Illkrich, France) according to the manufacturer’s instructions. Culture media were changed with fresh DMEM at 24 h after transfection and supernatants containing pseudovirus were harvested after 24 h of additional incubation.

For neutralization assay, Vero E6 cells were infected with each influenza A (H1N1) HA/NA- or SARS-CoV-2 spike-pseudotyped viruses. Before induction, pseudoviruses were incubated with λ-CGN for 2 h. Particularly, influenza A (H1N1) HA/NA pseudovirus was treated with 10 μg/ml of TPCK treated-trypsin for 15 min before λ-CGN treatment. After that, TPCK treated-trypsin was inactivated by 10 μg/ml Soybean solution (Sigma-Aldrich). Twenty four hours after infection, the medium was changed with fresh DMEM. Forty eight hours after transduction, BrightGlo (Promega, Madison, WI, USA) was added to the cell lysates according to the manufacturer’s instructions, and relative luminescence units (RLUs) were measured by a microplate reader.

### Quantitative RT-PCR

SARS-CoV-2-infected Vero cells (MOI, 0.005) were treated with increasing concentrations of λ-CGN or RDV at 37 °C. At day 2, culture supernatants were harvested for viral RNA purification using QIAamp viral RNA mini kit (Qiagen, Hilden, Germany). Relative SARS-CoV-2 RNA copies were quantified using a real-time RT-PCR kit with an N gene-specific primer set (PCLMD nCoV one step RT-PCR kit; PCL Inc., Seoul, Republic of Korea) and a CFX96 Touch real-time PCR instrument (Bio-Rad, Hercules, CA, USA).

### Statistical analysis

Statistical analyses were performed by unpaired, two-way ANOVA t-test according to the Dunnett’s multiple comparison method using GraphPad Prism version 8.3.1. In the Kaplan–Meier survival analysis, survival statistics were calculated by Log-rank (Mantel–Cox) test. *P* values lower than to 0.05 were considered statistically significant. *P < 0.05; **P < 0.01; ****P < 0.0001.

## Results

### Anti-influenza activity of λ-CGN

To examine the antiviral activity of λ-CGN, increasing concentrations of the compound were loaded to influenza virus-infected MDCK cells. Another sulfated polysaccharide, p-KG03, of which antiviral activity has been elucidated in our previous report^19^ and three different antiviral chemicals (AMT, RBV and OSV-C) were used as controls. The anti-influenza viral activity or the drug-resistance profiles of these control compounds were reproducible, indicating that the cell culture-based antiviral assay is reliable (Table 1). The cytopathic effect (CPE) inhibition assay revealed that λ-CGN efficiently inhibited infection by both influenza A and B viruses, with EC_50_ values of 0.3 to 1.4 μg/ml, with no cytotoxicity up to a maximum concentration of 300 μg/ml. Notably, the inhibitory effect was comparable with that of p-KG03. To confirm this finding, we measured changes in viral protein expression in cell lysates and infectious viral titers in culture supernatants (Fig. 1B,C, Supplementary Fig. S1). The data revealed that λ-CGN not only inhibited expression of viral proteins NP and HA in infected cells, but also suppressed production of progeny virus in a dose-dependent manner as observed in the p-KG03-treated samples. Taken together, these results suggested that λ-CGN has considerable antiviral activity against influenza A and B viruses in vitro, with selectivity index (SI) values over 263.2.

**Table 1:** Antiviral effect of λ-CGN against influenza A and B viruses.

Compound	CC_50_^a^	EC_50_^b^ (S.I.^c^)			Units
		PR8^d^	HK^e^	Lee^f^	
λ-CGN	> 300.0	0.3 ± 0.1 (> 1132)	0.3 ± 0.0 (> 1200)	1.4 ± 0.3 (> 214.3)	μg/ml
p-KG03	> 300.0	0.3 ± 0.1 (> 923.1)	0.4 ± 0.1 (> 770.2)	0.4 ± 0.0 (> 800.0)	μg/ml
AMT^g^	> 300.0	> 100.0 (N.D.^h^)	1.4 ± 0.5 (> 71.4)	> 100.0 (N.D.)	μM
RBV^i^	> 100.0	15.4 ± 0.3 (> 6.5)	11.9 ± 3.5 (> 8.4)	14.9 ± 0.4 (> 6.7)	μM
OSV-C^j^	> 100.0	0.02 ± 0.01 (> 5000)	< 0.005 (> 20,000)	1.09 ± 0.04 (> 91.7)	μM

^a^The half-maximal cytotoxic concentration.

^b^The half-maximal effective concentration.

^c^Selectivity index, the ratio of CC_50_ to EC_50_.

^d^A/Puerto Rico/8/34 (H1N1).

^e^A/Hong Kong/8/68 (H3N2).

^f^B/Lee/40.

^g^Amantadine hydrochloride.

^h^Not detected.

^i^Ribavirin.

^j^Oseltamivir carboxylate.

### Inhibition of influenza viral entry by λ-CGN

**Figure 2:**
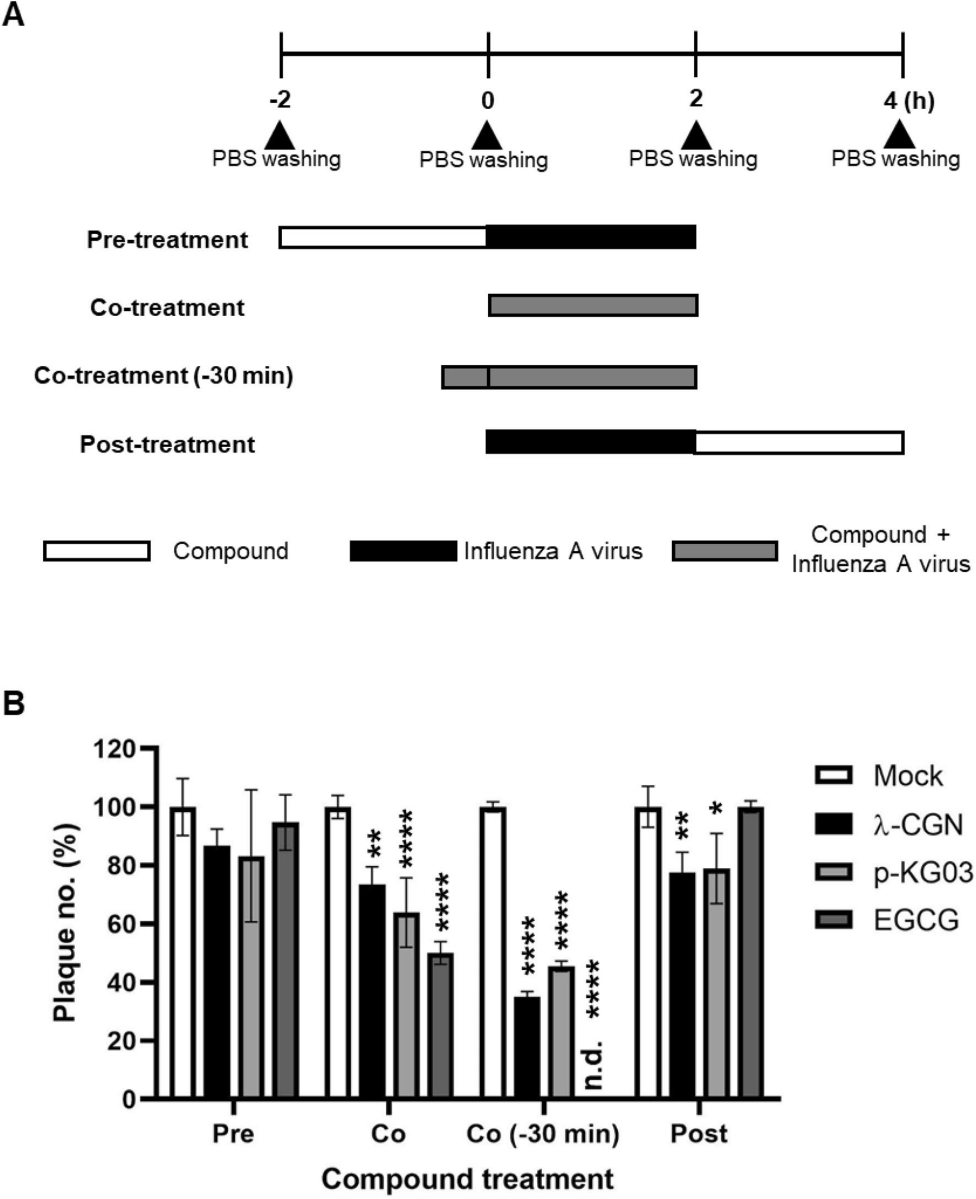
Time-of-addition experiment. (**A**) Schematic representation of compound treatment and virus infection. In the pre-treatment assay, MDCK cells were treated with λ-CGN (1 μg/ml) for 2 h and then infected with PR8 at an MOI of 0.001 for additional 2 h. In the co-treatment assay, the mixture of λ-CGN and PR8 was loaded onto the cells immediately or after 30-min-preincubation [Co-treatment (− 30 min)]. In the post-treatment assay, PR8-infected cells were added with λ-CGN for 2 h. As controls, p-KG03 (1 μg/ml) or EGCG (1 μM) was treated in all sets. In each step, cells were washed with PBS to remove non-specifically bound compounds or unabsorbed virus. (**B**) Plaque titration. The cells treated with compounds and PR8 at different time points were incubated in the overlay medium. At day 3 post-infection, viral plaques were counted in comparison to the mock-treated controls (set at 100%). Data are expressed as the mean ± S.D. of three independent experiments. *P < 0.05; **P < 0.01; ****P < 0.0001; n.d., not detected. Pre, pre-treatment of the compound; Co, co-treatment of the compound with virus; Co (− 30), co-treatment with 30 min-preincubated samples; post, post-treatment of the compound. The graph was created using GraphPad Prism 8.3.1 (www.graphpad.com).

We wondered which step of the influenza virus life cycle is targeted by λ-CGN. Using p-KG03 and EGCG as controls for blocking virus entry, λ-CGN was treated at different time points, such as before (pre-treatment), during (co-treatment) or after (post-treatment) viral infection at 2 h intervals (Fig. 2A). Plaque titration informed that its pre-treatment has little inhibitory effect, while post-treatment resulted in significant reduction but not remarkable (Fig. 2B). However, its co-treatment with the virus induced significant antiviral effect, that was more enhanced when they were pre-incubated for 30 min before treatment. All three compounds seem to target viral entry step by directly attenuating infectivity of the viral particles. The result suggested that λ-CGN could interact with a viral protein important for virus entry, possibly HA.

**Figure 3:**
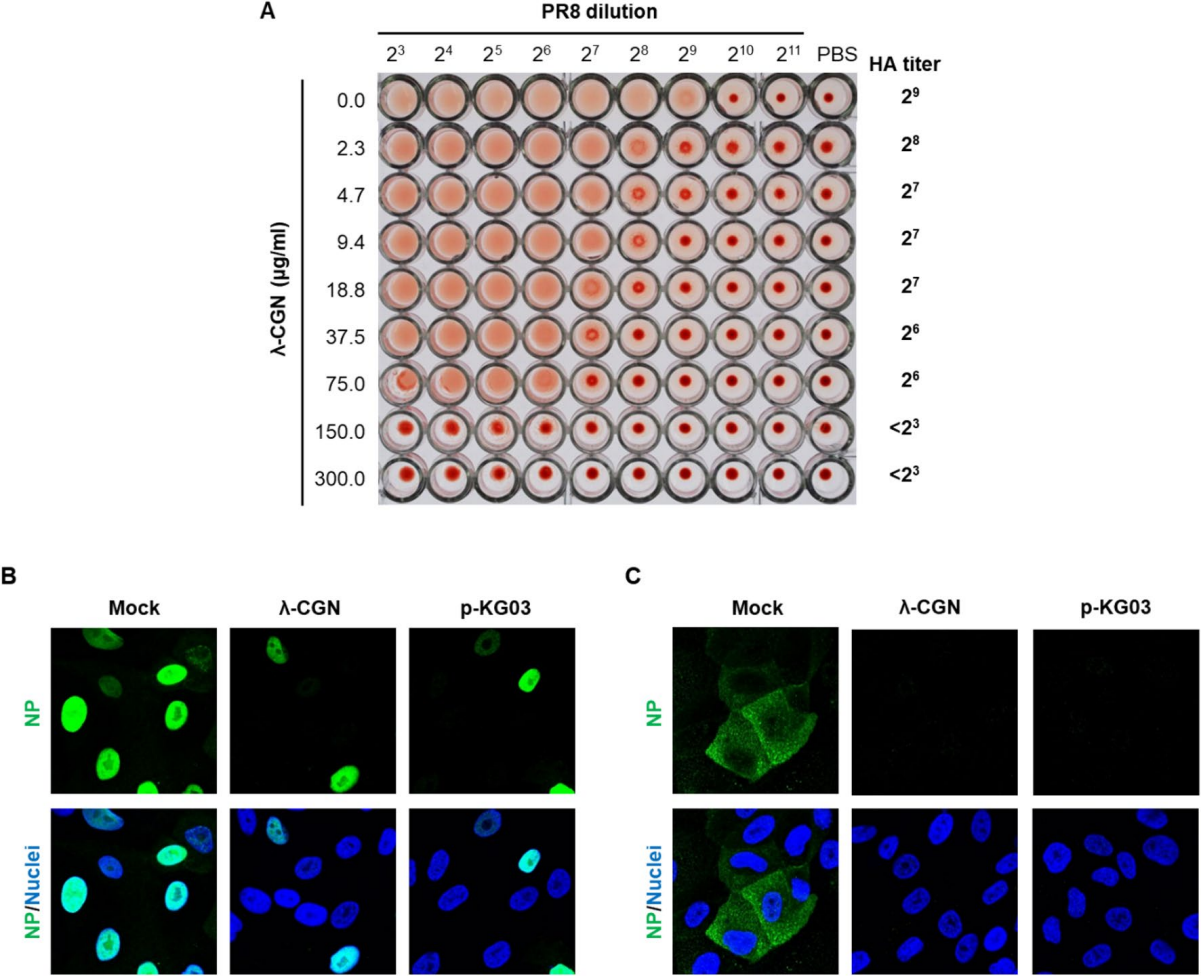
Effect of λ-CGN on the influenza A virus entry. (**A**) HA inhibition assay. Two-fold serially diluted PR8 (from 2^3^ to 2^11^) in PBS was incubated with an equal volume of PBS or twofold increasing concentrations of λ-CGN for 20 min. HA titer in each combination was determined at 30 min after addition of 0.5% chicken RBC. HA titers are marked on the right side of the panel. (**B**,**C**) Confocal microscopy. MDCK cells were infected with PR8 (MOI, 5) in the absence (Mock) or presence of either λ-CGN or p-KG03 at a concentration of 10 μg/ml. At 4 h post-infection in the absence of CHX (**B**) or at 2.5 h in the presence of 10 μg/ml CHX (**C**), viral NP was detected with an anti-NP antibody and an Alex Fluor 488-conjugated goat anti-mouse secondary antibody (green). Cell nuclei were counterstained with DAPI (blue). Original magnification, 400×. The images were analyzed using ZEN blue software 3.1 (www.zeiss.com).

To evaluate this hypothesis, we examined HA inhibition assay with λ-CGN (Fig. 3A). Agglutination of chicken RBC was achieved by PR8 at the highest dilution 2^8^. However, HA titer was gradually reduced as the concentration of λ-CGN was increased, confirming that it inhibits binding of viral HA to cellular receptors. To further verify its mode-of-action, intracellular distribution of viral NP was compared in the absence or presence of the compound at 4 h p.i, a time when NP was fully localized to the nuclei for robust replication of viral RNA (Fig. 3B, Supplementary Fig. S2). The confocal microscopic images revealed that, similar to p-KG03, λ-CGN reduced the number of NP-positive nuclei when compared to the mock-treated sample. We also monitored the intracellular distribution of NP at an earlier time point (2.5 h post-infection) in the presence of CHX, a protein synthesis inhibitor that allows tracking of the input viral proteins and their localization (Fig. 3C). Under this condition, when NP was present in the cytoplasm but not reached the nucleus, λ-CGN completely blocked membrane penetration of the viral particles harboring vRNP complexes as efficiently as p-KG03. No NP accumulation on the surface of the cellular membrane strongly suggests that λ-CGN targets attachment of influenza virus to its cell surface receptors by neutralizing viral glycoprotein HA.

### Protection of mice from influenza A viral infection by λ-CGN

**Figure 4:**
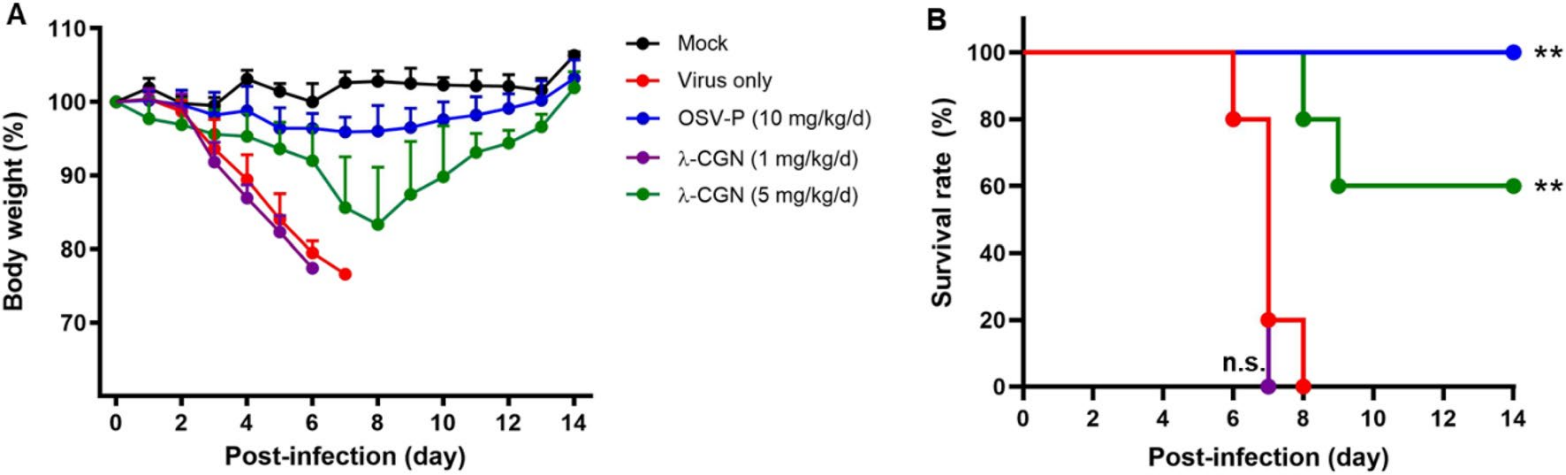
Antiviral effect of λ-CGN against influenza A virus in vivo. BALB/c mice (6–7 weeks old female) were mock-infected (black) or intranasally infected with maPR8 at 5 MLD_50_ (red). As test groups, the virus was preincubated at room temperature for 30 min with λ-CGN at a lower dose (1 mg/kg/d, purple) or a higher dose (5 mg/kg/d, green), followed by intranasal administration. Control mice received OSV-P orally twice a day (10 mg/kg/d) at 8-h intervals, starting at 4 h before viral infection (blue). Body weight (**A**) and mortality (**B**) of mice were measured every day from days 0 to 14 post-infection. Data are expressed as the mean ± S.D. from five mice. Survival statistics were calculated by Log-rank (Mantel–Cox) test. **P < 0.01; n.s., non-statistically significant. The graphs were created using GraphPad Prism 8.3.1 (www.graphpad.com).

To investigate the antiviral activity of λ-CGN in vivo, mice were infected intranasally with maPR8 alone or with maPR8 plus λ-CGN once. As a control, maPR8-infected mice received OSV-P orally twice a day for 6 days. Antiviral activity was determined by monitoring body weight and mortality for 15 days. The results revealed that maPR8 at 5 MLD_50_ caused body weight loss (Fig. 4A) and complete death within day 7 (Fig. 4B). Notably, intranasal administration of 5 mg/kg λ-CGN mitigated infection-mediated body weight loss, yielding a 60% survival rate. However, this antiviral efficacy was not observed at a lower dose (1 mg/kg). As expected, treatment with OSV-P at 10 mg/kg/day for 6 days showed remarkable therapeutic effects, ensuring the reliability of the in vivo antiviral study. Taken together, these data suggested that intranasal co-administration of λ-CGN prevents viral infection-mediated body weight loss and reduces mortality.

### Anti-SARS-CoV-2 activity of λ-CGN

**Figure 5:**
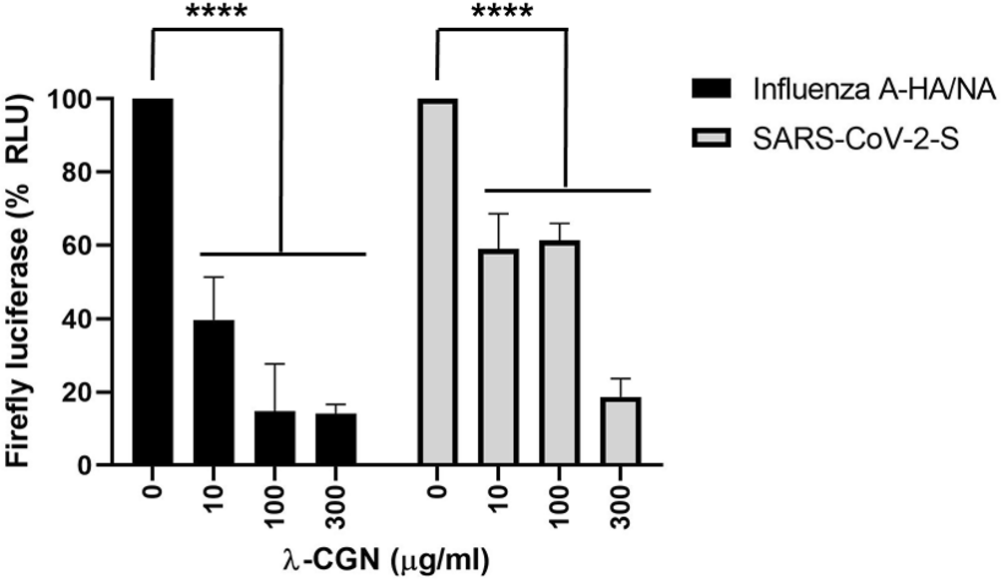
Inhibition of infection by influenza A virus (H1N1) HA/NA- or SARS-CoV-2 spike-pseudotyped viruses. Lentiviral pseudotypes bearing influenza A virus (H1N1) HA and NA proteins (black bars) or SARS-CoV-2 spike protein (gray bars) were prepared, in which a firefly luciferase-expressing plasmid was incorporated. They were preincubated with mock or increasing concentrations of λ-CGN for 2 h at 37 °C and then infected into Vero E6 cells. On day 2, relative firefly luciferase activity (RLU) was determined by fixing the mock-treated sample at 100%. Data are expressed as the mean ± S.D. of three independent experiments. ****P < 0.0001. The graph was created using GraphPad Prism 8.3.1 (www.graphpad.com).

**Figure 6:**
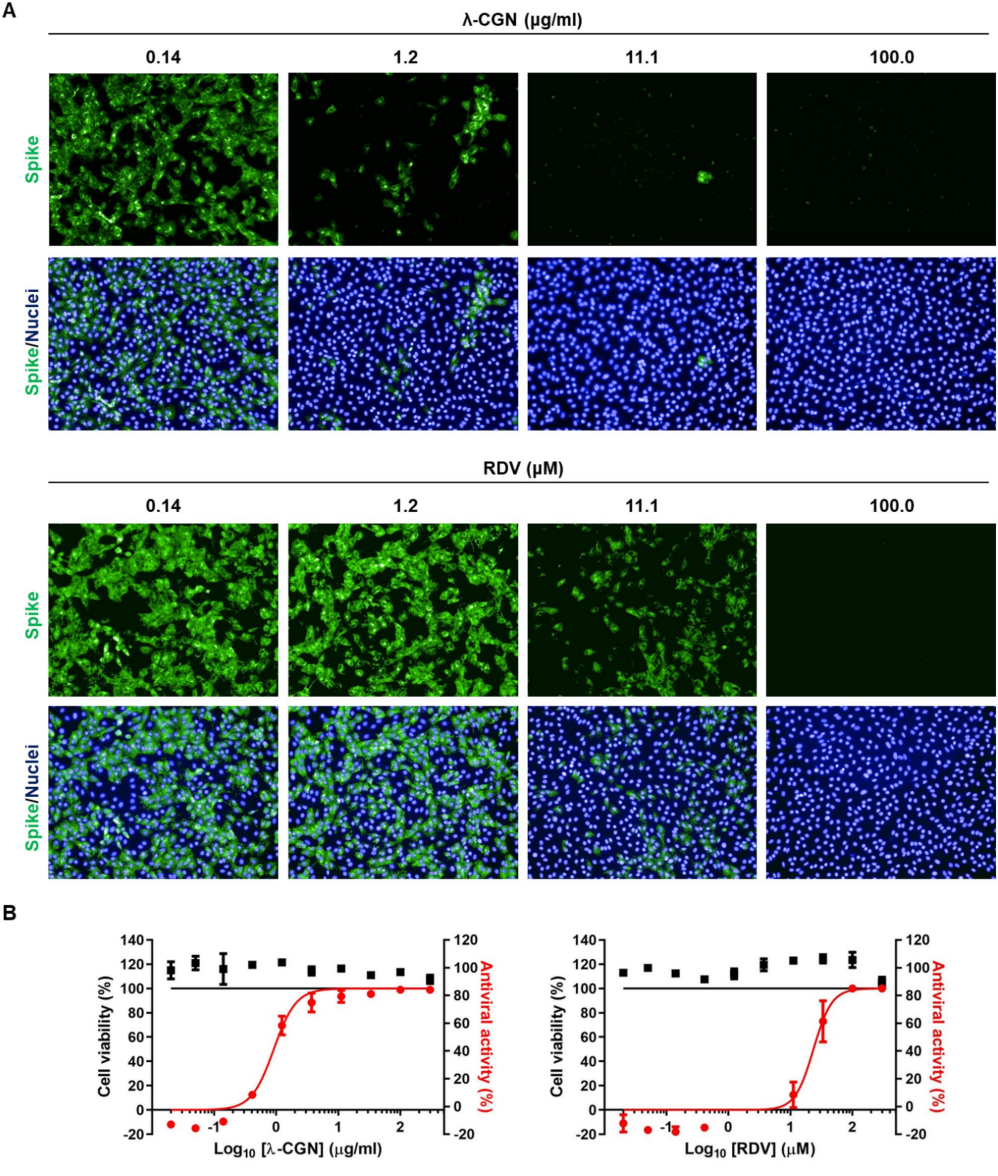
Image-based antiviral analysis of λ-CGN against SARS-CoV-2. (**A**) Vero cells seeded in 96-well plates were infected with SARS-CoV-2 at an MOI of 0.02, either alone or in the presence of increasing concentrations of λ-CGN (upper panel) or RDV (lower panel; a control). On day 2 post-infection, cells were fixed and permeabilized prior to immunostaining with an anti-SARS-CoV-2 spike antibody and an Alexa Fluor 488-conjugated goat anti-mouse IgG (green). Cell nuclei were counterstained with DAPI to estimate cell viability (blue). Images were captured with a 20× objective lens fitted to an automated fluorescence microscope by using the Harmony High-Content Imaging and Analysis software 3.5.2 (www.perkinelmer.com). (**B**) The number of fluorescent green and blue spots was counted to calculate antiviral activity (red circles) and cell viability (black squares), respectively, at each concentration of the compounds. The viability of mock-infected cells were fixed as 100%, while the antiviral activity in virus-infected cells or mock-infected cells was fixed as 0 and 100%, respectively. Data are expressed as the mean ± S.D. from three independent experiments. The graphs were created using GraphPad Prism 8.3.1 (www.graphpad.com).

**Figure 7:**
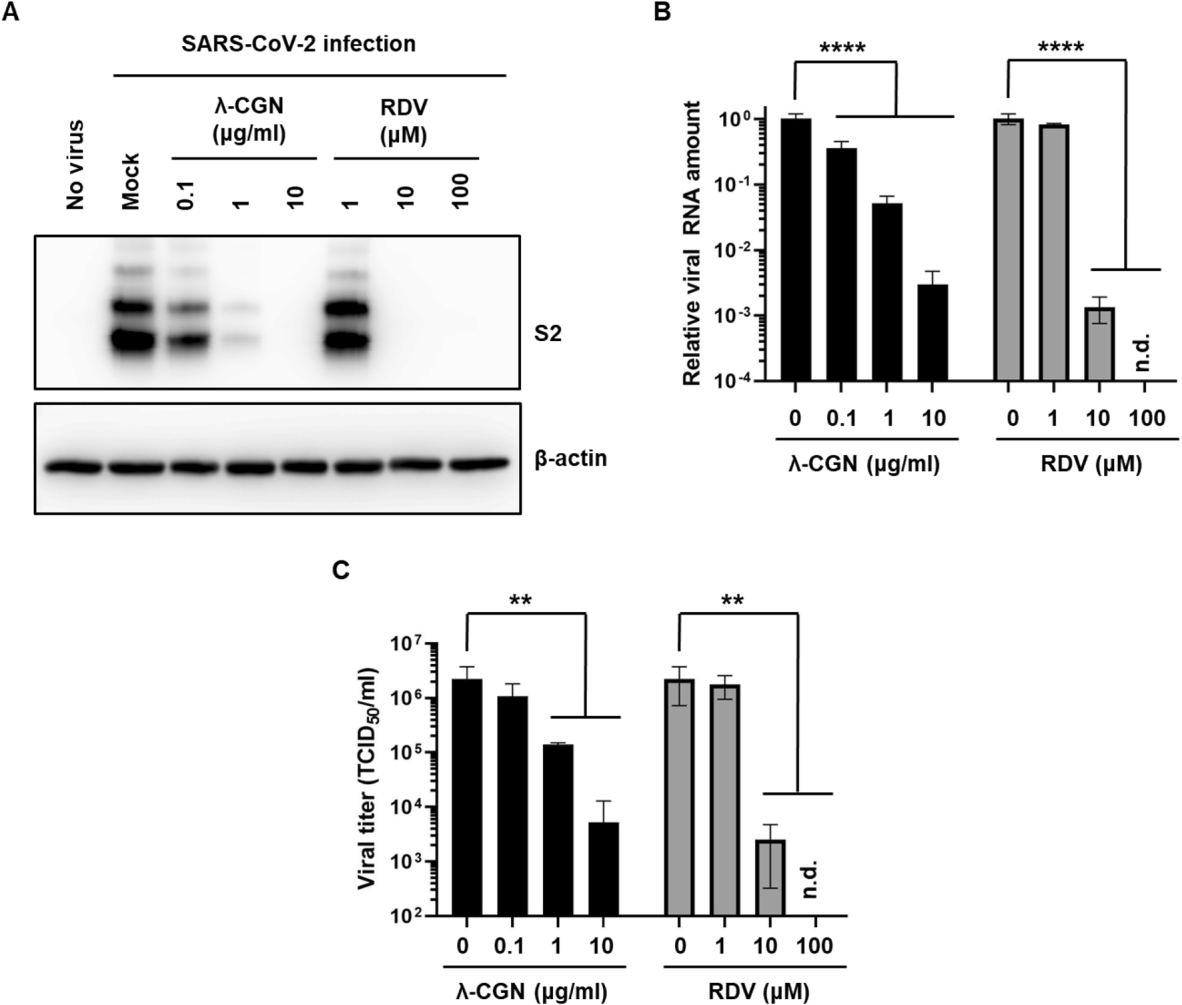
Anti-SARS-CoV-2 activity of λ-CGN. (**A**) SARS-CoV-2-infected Vero cells (MOI, 0.005) were treated with λ-CGN or RDV for 2 days. Whole cell lysates were subjected to immunoblotting with anti-SARS-CoV-2 spike antibody (upper panel), in which β-actin was used as a loading control (lower panel). Proteins are marked on the right side of the panels. No virus, cell lysates without SARS-CoV-2 infection; Mock, SARS-CoV-2-infected cell lysates without antiviral compound treatment. S2, S2 subunit of viral spike protein. (**B**) Viral RNA was purified from the culture supernatants of the samples as mentioned in (**A**). Real-time RT-PCR was conducted using SARS-CoV-2 N gene-specific primers. Relative viral RNA copies were calculated on the basis of their Cq values. (**C**) Culture supernatants used in (**B**) were serially diluted for infection of fresh Vero cells. On day 2, the cell culture plates were subjected to immunofluorescence assay for determination of infectious viral titer by calculating the relative ratio of spike-derived green fluorescence frequency to nuclei-derived blue-positive cell number. Data are expressed as the mean ± S.D. of three independent experiments. **P < 0.01; ****P < 0.0001; n.d., not detected. The graphs were created using GraphPad Prism 8.3.1 (www.graphpad.com).

To address whether λ-CGN can neutralize SARS-CoV-2 as well as influenza virus, SARS-CoV-2 spike- and influenza A (H1N1) HA/NA-pseudotyped viruses bearing a firefly luciferase reporter gene were prepared^23^. Firefly luciferase assay with their infected cell lysates revealed that λ-CGN is able to suppress entry of both SARS-CoV-2 and influenza A viral glycoproteins-derived pseudoviruses in a dose-dependent manner (Fig. 5). As a next step, we examined antiviral activity of the sulfated polysaccharide against infectious SARS-CoV-2. Vero cells infected with the virus at an MOI of 0.02 were treated with increasing concentrations of λ-CGN by using RDV as a control. On day 2, immunofluorescence microscopy with an anti-SARS-CoV-2 spike antibody revealed that viral infection was inhibited effectively by λ-CGN, without affecting cell viability (Fig. 6A). As expected, anti-SARS-CoV-2 activity was well induced in the RDV-treated cells. Quantitative analysis of antiviral dose–response and cell viability showed that λ-CGN had an EC_50_ of 0.9 ± 1.1 μg/ml and a CC_50_ of > 300.0 μg/ml (resulting in an S.I., > 333.3), while RDV had an EC_50_ of 23.5 ± 1.2 μM and a CC_50_ of > 300.0 μM (resulting in an S.I., > 12.8) (Fig. 6B). In addition, western blot and quantitative RT-PCR analyses exhibited decreases of both viral protein in cell lysate and viral RNA level in the culture supernatants by the sulfated polysaccharide (Fig. 7A,B, Supplementary Fig. S3). Decisively, reinfection of the culture supernatants into fresh Vero cells confirmed reduction of infectious viral titers in the presence of λ-CGN as observed in RDV (Fig. 7C). These results strongly demonstrate that λ-CGN is active against SARS-CoV-2.

## Discussion

Sulfated polysaccharides such as heparin, dextran sulfate, and pentosan sulfate, as well as various CGNs, show antiviral or virucidal activity against diverse enveloped viruses at subtoxic concentrations^24–28^. These studies of the physiochemical properties and molecular structure of these compounds reveal that the antiviral efficacy is mainly due to their affinity for viral glycoproteins, resulting in blockade of viral attachment to cellular receptors. Accordingly, it is convincing that the charge density, chain length, degree of sulfation, and detailed structural features of these macromolecules are critical for this interaction. In-depth studies of the underlying mechanisms demonstrated that the macromolecules exert anti-HIV activity by competing with polyanionic regions of host-cell-surface molecules for binding to the positively charged amino acids present in the viral enveloped glycoprotein, gp120, including the V3 loop^29–31^. The microbicidal activity of polystyrene sulfonate against sexually transmitted infectious diseases caused by herpes simplex virus type 2 and papillomavirus has been evaluated in vivo and in vitro^32,33^. Unfortunately, prevention of vaginal HIV transmission using topical cellulose sulfate gel failed^34^, indicating the need for development of a more potent microbicidal sulfated polysaccharide or for administration of the polymers through an alternative route, such as oral or intranasal.

Regarding this issue, it is not strange to anticipate that intranasal treatment with active sulfated polysaccharides could be a promising way to prevent infection by various respiratory enveloped viruses such as influenza A and B viruses, respiratory syncytial virus, and coronaviruses. Previously, it was reported that κ-CGN with a molecular weight of 2 kDa is active against influenza A virus in vitro, with an EC_50_ value of 32.1 μg/ml. In addition, ι-CGN inhibited influenza A virus infection of MDCK cells with an EC_50_ value of 0.04–0.20 μg/ml; not only that, intranasal administration of ι-CGN showed therapeutic effects in an influenza A virus-infected mouse model^4,35^. Notably, a randomized double-blind study in volunteers with early symptoms of the common cold confirmed the efficacy and safety of an antiviral ι-CGN nasal spray^36^. In contrast to κ- and ι-CGNs, the antiviral activity of λ-CGN has rarely been investigated in the context of viral species that are transmitted in droplets or through the air. Therefore, we explored whether λ-CGN is able to inhibit both influenza A and B viruses and/or the emerging SARS-CoV-2. We had a great interest in λ-CGN, because this compound comprising alternating (1,3)-linked α-D-galactose-2-sulfated and (1,4)-linked β-D-galactose-2,6,-disulfated units has a higher degree sulfation with an ester sulfate content of about 32–39% and shows better solubility in cold water than the other two CGNs^37^. Accordingly, the sulfated polysaccharide was expected to have efficient and broad antiviral activity and to be easily dissolved in an aqueous solution when it is formulated for a nasal spray.

Here, we successfully observed that λ-CGN inhibits not only influenza viruses but also SARS-CoV-2 by targeting their entry process. Strikingly, its virucidal properties led to a 60% survival rate in virus-challenged mice after an exposure of infectious virus to the antiviral agent (Fig. 4). However, it remains to be investigated whether this polyanionic compound is able to protect small animals such as hACE2-expressing mice or Syrian hamsters from SARS-CoV-2 infection by blocking the viral S protein-associated entry step^38,39^. In addition, because CGNs have intrinsic anti-coagulant activity, any unwarranted side effects should be reviewed before clinical application. This is because dysfunctional or aberrant coagulation is responsible for the hyper-inflammatory responses observed in severe cases of influenza or SARS-CoV-2 infection-mediated pneumonia, and anti-coagulant signals could be over-stimulated already in the lungs of infected patients^40,41^. Nevertheless, to the best of our knowledge, this is the first report to suggest that λ-CGN potently inhibits infection by influenza B as well as influenza A viruses and emerging SARS-CoV-2. The broad spectrum antiviral activity of this compound would make it valuable especially when different respiratory viruses are circulating concurrently or when their prophylactic treatment is definitely required before diagnosis.

## Supplementary Information

Supplementary Figures.
