# Supplementary material for: Antiviral activity of lambda-carrageenan against influenza viruses and severe acute respiratory syndrome coronavirus 2
Source: Sci Rep. 2021 Jan 12;11:821. doi: 10.1038/s41598-020-80896-9 (PMC7804421; doi:10.1038/s41598-020-80896-9)
Supplement: Supplementary file 1 — Supplementary Figures. [file 41598_2020_80896_MOESM1_ESM.pdf]

Antiviral activity of lambda-carrageenan against influenza viruses and severe acute respiratory syndrome coronavirus 2

Yejin Jang[1], Heegwon Shin[2], Myoung Kyu Lee[1], Oh Seung Kwon[1,†], Jin Soo Shin[1], Yongil Kim[3], Chan Woo Kim[4], Hye-Ra Lee[4] & Meehyein Kim[1,5,*]

[1]Infectious Diseases Therapeutic Research Center, Korea Research Institute of Chemical Technology (KRICT), Daejeon 34114, Republic of Korea

[2]Department of Chemistry, Korea Advanced Institute of Science and Technology (KAIST), Daejeon 34141, Republic of Korea

[3]Hanmi Pharmaceutical Co., Hwaseong-si, Gyeonggi-do 18536, Republic of Korea

[4]Department of Biotechnology and Bioinformatics, College of Science and Technology, Korea University, Sejong 30019, Republic of Korea

[5]Graduate School of New Drug Discovery and Development, Chungnam National University, Daejeon 34134, Republic of Korea

*** Corresponding author**

Meehyein Kim, Ph.D.

Infectious Diseases Therapeutic Research Center, Korea Research Institute of Chemical Technology, 141 Gajeongro, Yuseong, Daejeon 34114, Republic of Korea

E-mail: mkim@krict.re.kr; Telephone: 82-42-860-7540

[†] Current address: siRNAgen Therapeutics Co., Daejeon 34302, Republic of Korea

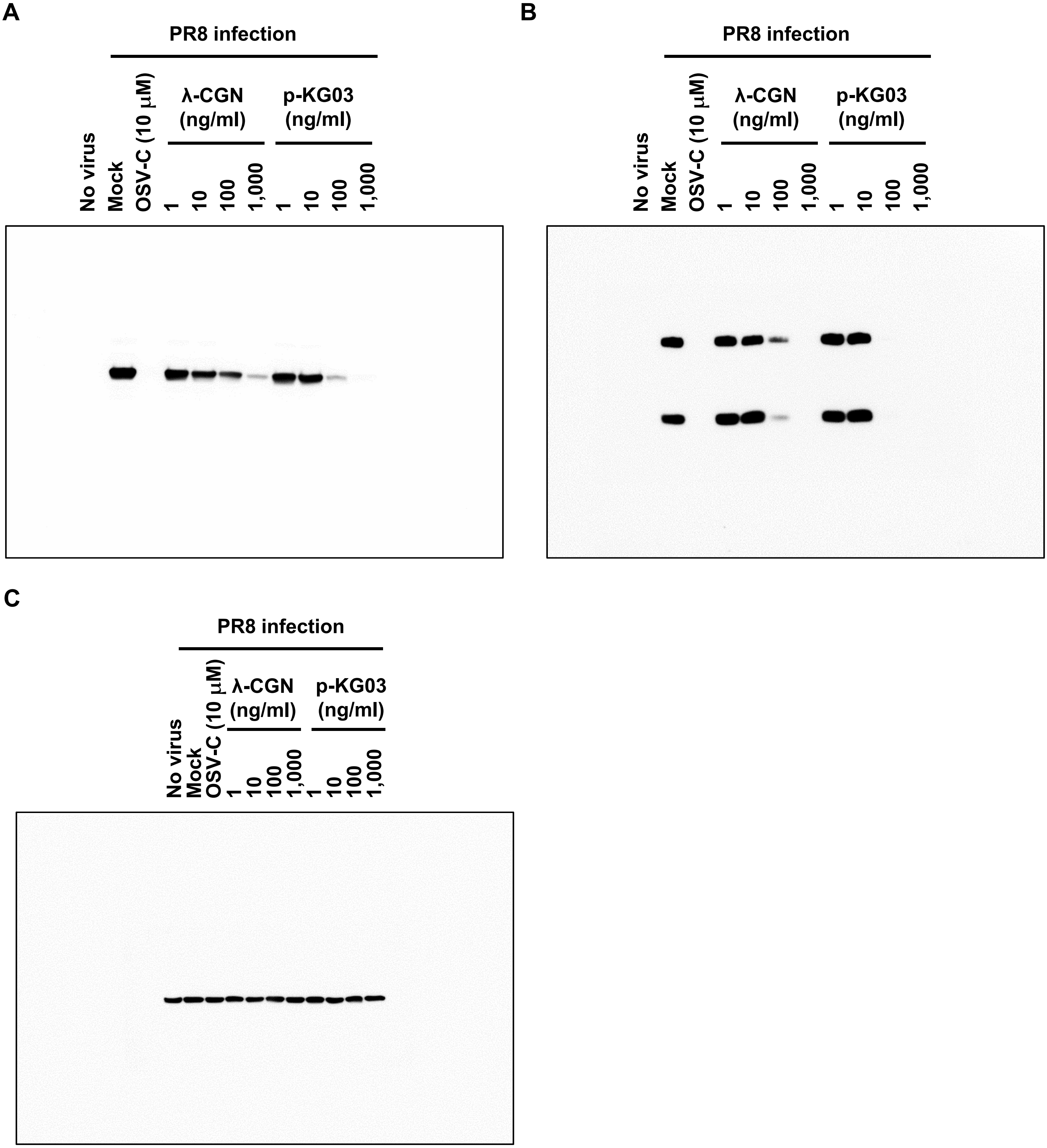

Supplementary Figure S1. Raw data showing full images of the western blots presented in Fig. 1B. MDCK cells infected with PR8 at an MOI of 0.001 were mock-treated (Mock) or treated with increasing concentrations of λ-CGN or p-KG03, or with 10 μM of OSV-C, at 35℃. On the next day, cell lysates were harvested for SDS-PAGE and immunoblotting with anti-NP (A) or anti-HA antibodies (B). β-Actin was used as a loading control (C). 'No virus' means negative control without viral infection. HRP on the membranes for detecting NP and HA was developed using Western Femto ECL kit (LPS Solution, Daejeon, Republic of Korea) (A, B), while it was done for β-actin using Super Signal West Pico Plus Chemiluminescent kit (Thermo Fisher Scientific, Rockford, IL, USA) (C).

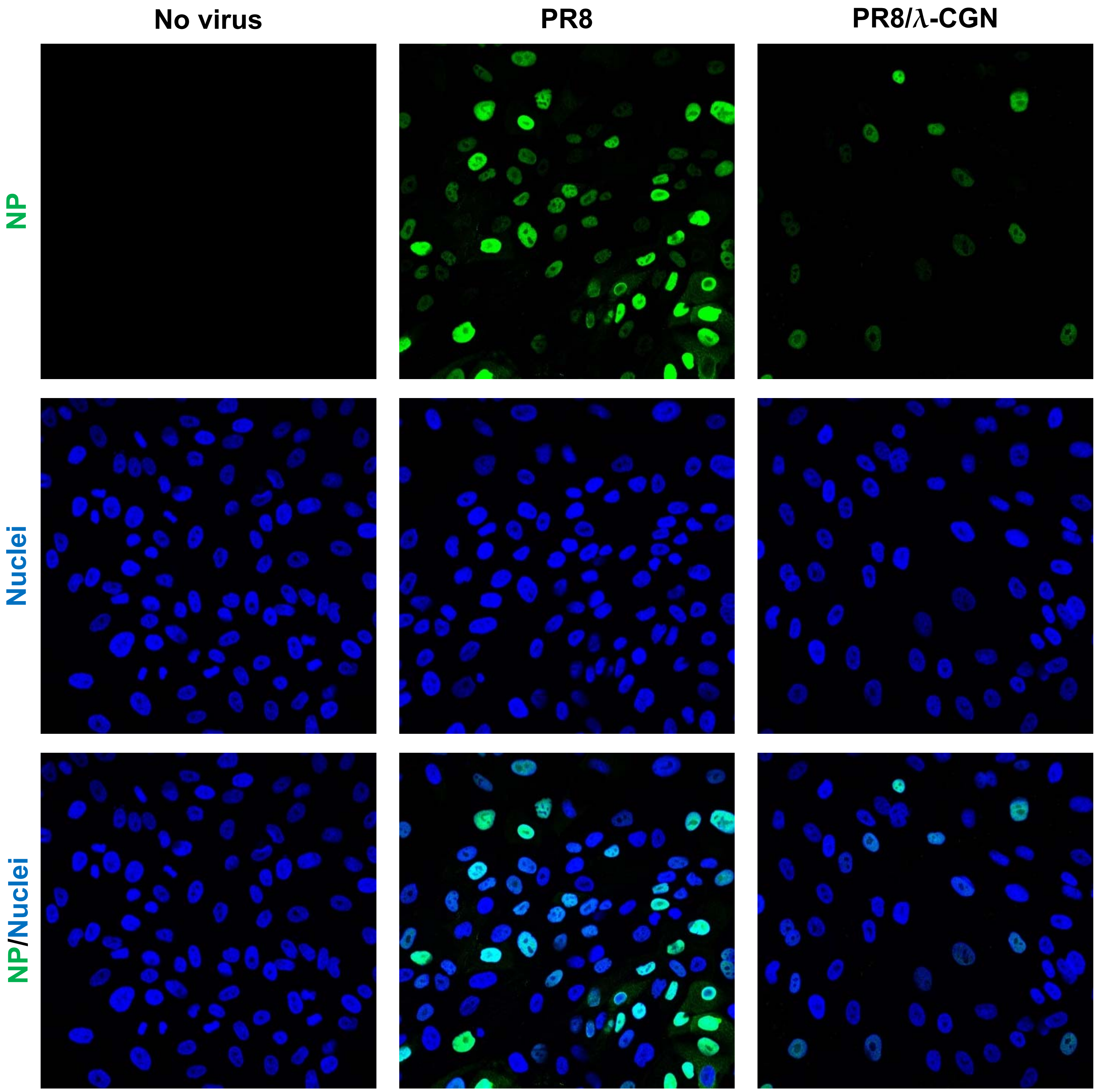

Supplementary Figure S2. Wider field images of confocal microscopy. MDCK cells were mock-infected (No virus) or infected with PR8 (MOI, 5). Infected cells were treated with λ-CGN at a concentration of 10 μg/ml. At 4 h post-infection, viral NP was detected with an anti-NP antibody and an Alex Fluor 488-conjugated goat anti-mouse secondary antibody (green). Cell nuclei were counterstained with DAPI (blue). Original magnification, 400×.

A

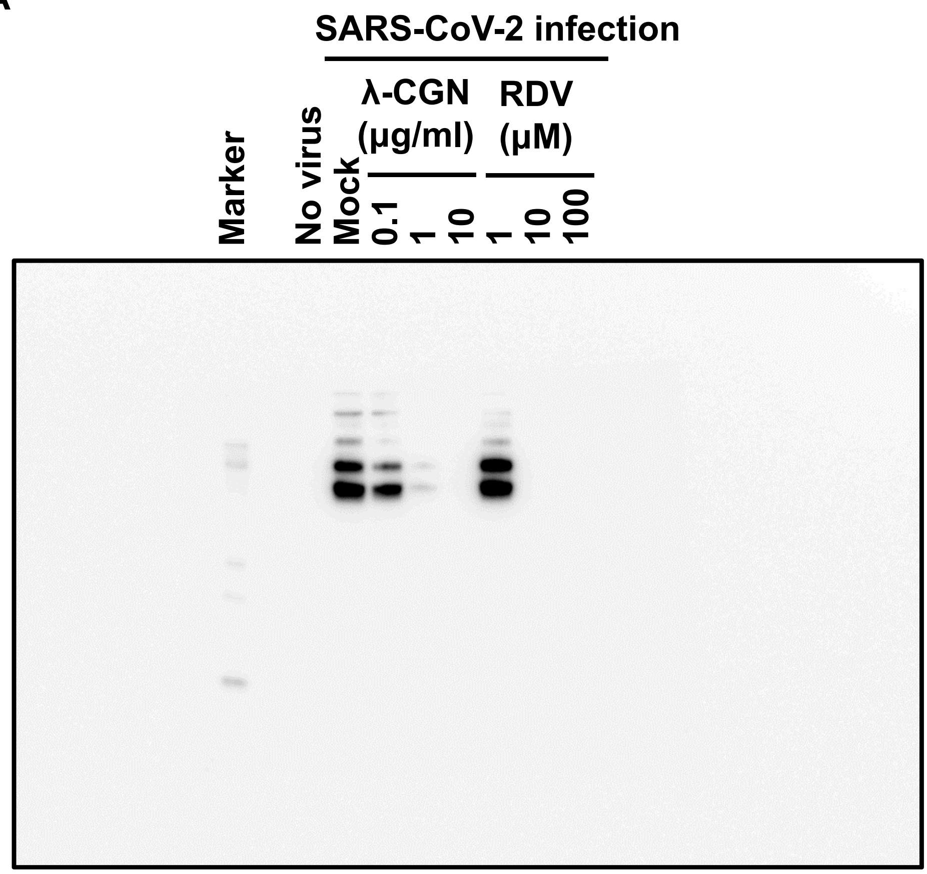

B

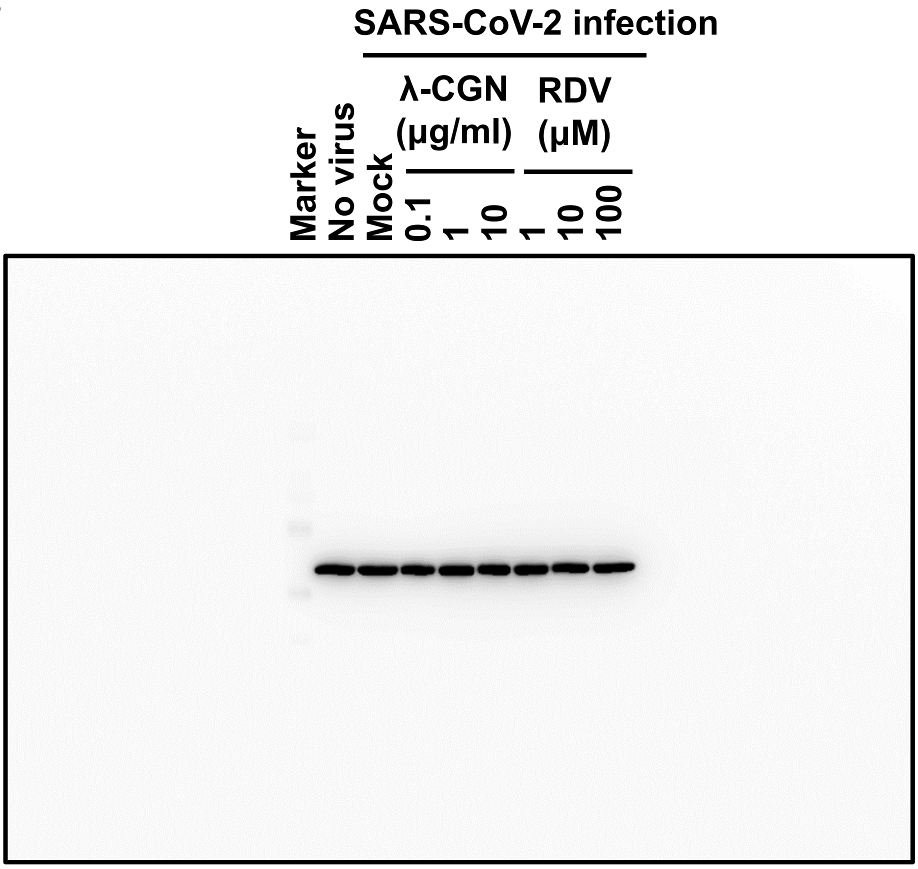

Supplementary Figure S3. Raw data showing full images of the western blots presented in Fig. 7A. Vero cells infected with SARS-CoV-2 at an MOI of 0.005 were mock-treated (Mock) or treated with increasing concentrations of λ-CGN or remdesivir (RDV) at 37°C. On day 2, cell lysates were harvested for SDS-PAGE and immunoblotting with anti-spike antibody (A) or anti-β-actin as a loading control (B). 'Marker', protein size markers. 'No virus', a negative control without viral infection.
